# A scoping review of endoscopic and robotic techniques for lateral neck dissection in thyroid cancer

**DOI:** 10.3389/fonc.2024.1297972

**Published:** 2024-02-08

**Authors:** Alexis L. Woods, Michael J. Campbell, Claire E. Graves

**Affiliations:** Department of Surgery, University of California, Davis Medical Center, Sacramento, CA, United States

**Keywords:** endocrine surgery, thyroid cancer, lateral neck dissection, endoscopic lateral neck dissection, remote access lateral neck dissection, video-assisted lateral neck dissection, robotic lateral neck dissection

## Abstract

**Introduction:**

Lateral neck dissection (LND) in thyroid cancer has traditionally been performed by a transcervical technique with a large collar incision. With the rise of endoscopic, video-assisted, and robotic techniques for thyroidectomy, minimally invasive LND is now being performed more frequently, with better cosmetic outcomes.

**Methods:**

The purpose of this paper is to review the different minimally invasive and remote access techniques for LND in thyroid cancer. A comprehensive literature review was performed using PubMed and Google Scholar search terms “thyroid cancer” and “lateral neck dissection” and “endoscopy OR robot OR endoscopic OR video-assisted”.

**Results:**

There are multiple surgical options now available within each subset of endoscopic, video-assisted, and robotic LND. The approach dictates the extent of the LND but almost all techniques access levels II-IV, with variability on levels I and V. This review provides an overview of the indications, contraindications, surgical and oncologic outcomes for each technique.

**Discussion:**

Though data remains limited, endoscopic and robotic techniques for LND are safe, with improved cosmetic results and comparable oncologic and surgical outcomes. Similar to patient selection in minimally invasive thyroidectomy, it is important to consider the extent of the LND and select appropriate surgical candidates.

## 1 Introduction

The incidence of thyroid cancer has been increasing over time, with differentiated cancers comprising 95% of thyroid tumors (1, 2). Papillary thyroid cancer (PTC) has been shown to metastasize to lymph nodes (LN) in up to 30% of cases, and with micrometastasis rates of up to 80% (1, 3). Follicular thyroid cancer has a much lower rate of LN metastasis, less than 5%, with Hürthle cell thyroid cancer rates between 5-13%. In the most recent guidelines from the American Thyroid Association, indications for performing a therapeutic selective lateral neck dissection (SLND) are the presence of LN metastases (LNM) in the lateral compartment proven by thyroglobulin washout and/or biopsy (4). The extent of SLND, however, is still debated. There has been extensive discussion in the literature on the pattern of metastatic LN spread to levels of the neck and which levels should be included in a SLND (1, 5–11). NCCN 2023 guidelines recommend SLND should be comprised of level II-IV, Vb in the presence of lateral LNM, with the addition of level I or Va if clinically involved (12). LNM are thought to typically follow the pattern of spread to the central neck in level VI (+/- VII), followed by ipsilateral spread to the lateral neck (1). In the lateral compartment, the first level to be affected most often is level IV, then III, II and V in that order (1, 6). While a lateral neck dissection (LND) is typically only recommended for clinically evident lateral LNM, occult metastases to the lateral cervical nodes have been found in up to 38-90% of patients with clinically negative cervical LN (7). Skip metastases have also been found in up to 21% of patients in a study by Park et al, most-often associated with upper pole tumors (13, 14). Kim et al. similarly found skip LNM in 28.2% of 241 patients with PTC who underwent a total thyroidectomy, central node dissection (CND), and SLND (levels II-IV) (5). Previous papers have suggested that level I, II, and V do not need to be included in LND unless clinical or radiologic evidence of LNM in those levels (10). In Javid et al’s retrospective review of 202 modified radical neck dissections (MRND), they found that omitting levels II potentially misses level II disease in two-thirds of patients, and omission of level V misses level V disease in one-fifth of patients (11). Multiple studies have also advocated for inclusion of level V in all SLND even if not clinically involved pre-operatively due to the poor sensitivity of ultrasound or computerized tomography scan detecting level V LNM (<30%) (6, 9). Given that patients with lateral neck disease have a 6-fold increased chance of recurrence compared with patients without LNM, some institutions perform MRND for all patients with clinically-evident lateral neck disease, which include level I-V, with preservation of one or more non-lymphatic structures, such as the sternocleidomastoid muscle (SCM), spinal accessory nerve, and internal jugular vein (IJV) (15–17). Because of the risk of perioperative morbidity and change in quality of life from MRND, including injury to the spinal accessory nerve, spinal roots, or marginal mandibular nerve, other institutions perform less extensive SLND of variable levels beyond level III-IV.

The conventional LND requires a long collar or L-shaped scar that can greatly impact the quality of life of patients, particularly in young patients (18). With the rise of minimally invasive and remote access approaches to thyroid surgery over the last 25 years, many of these are now being performed for LND, especially in Asian countries. The adoption of many remote access techniques has been slower in North America and Europe (19, 20). Multiple reasons for this have been posited, including different body habitus, disease characteristics, and a stronger cultural stigma against an anterior neck scar in Asia (19–23). Additionally, other reasons include the higher cost, learning curve, longer operative times, and argument that many remote access techniques are actually more invasive with a larger dissection required (24). Despite global differences in practice, endoscopic and robotic thyroidectomies have been shown to be safe with similar surgical completeness and oncologic outcomes, as well as superior cosmetic outcomes (20, 21, 25, 26). The extent of SLND varies depending on the minimally invasive or remote access approach. In this review we will discuss video-assisted approaches, endoscopic remote access, and robotic remote access techniques, including an overview of the technique, indications, contraindications, and surgical and oncologic outcomes for each technique.

## 2 Methods

This review was performed in accordance with Preferred Reporting Items for Systematic reviews and Meta-Analyses extension for Scoping Reviews (protocol not applicable) (27). A comprehensive literature review was performed in June 2023 using PubMed and Google Scholar search terms “thyroid cancer” and “lateral neck dissection” and “endoscopy OR robotic OR endoscopic OR video-assisted” written in English. Search results yielded 85 papers in PubMed and 375 in Google Scholar, for a total of 460 papers (for flow chart see **Supplementary Figure 1**). Studies comprised of thyroid cancer patients who underwent lateral neck dissections by either video-assisted, endoscopic, or robotic techniques were included. Duplicate records were removed (n = 43). Conference oral or poster abstract, book chapters, textbooks, studies that discussed pediatric cases, other organ systems or cancers other than thyroid cancer were removed (n = 300). Of the papers screened (n = 117), 61 were excluded for not including LND in their dissection, were descriptive only, or they did not present data for patients who underwent remote access LND separately. Of 56 papers sent for retrieval, 5 were not retrievable. For studies with overlapping patient cohorts (n = 2), the study with the highest quality and largest sample size was selected for data analysis. Case series of a remote access technique or comparative studies between a remote access technique and conventional LND were included (n = 47). One investigator reviewed and screened abstracts and full-text articles by using the above inclusion and exclusion criteria. Evaluation of methodological quality of the studies was performed utilizing Methodological Index for Non-Randomized Studies (MINORS) criteria (28). Of the 47 studies evaluated, 21 were deemed poor (case series score ≤ 8 or comparative study score ≤ 14), 26 were moderate (case series score 9-14 or comparative study score 15-22) (**Supplementary Table 1**). All studies were included in the review due to scarcity of data on remote access techniques. Data was extracted to include sample size, extent of LND, operative time, length of hospital stay, lymph node yield, surgical complications, length of follow-up, number of recurrences, and cosmetic satisfaction scores (**Tables 1**–**9**). In studies with heterogenous groups, where only a subset of patients underwent remote access LND, only data reported separately for malignant cases that underwent LND were extracted. If the surgical outcome data was not presented separately, not applicable (N/A) was used in the tables. In studies where mean follow up time was given in days rather than months, it was converted to months by a denominator of 30. To create uniform language, if surgical complications were reported as transient or permanent hypocalcemia, this was referenced as transient or permanent hypoparathyroidism. Similarly, if different language was used other than transient or permanent recurrent laryngeal nerve (RLN) palsy, this was standardized. If the remote access thyroidectomy and LND study group had variation in the incisions or approach used, this was clarified in the text and tables (28).

**Table 1:** Video-Assisted Anterior Neck Approach.

Author (Year of publication; citation number)	Study Design/ Approach	No. of Cases	Mean Operative Time (min)	Levels Dissected	Lymph Node Yield Mean (range)	Mean Length of Stay in Days (range)	Complication (cases)	Mean Follow up (months) and recurrence
Ikeda et al (2002, 29)	Case series/ Neck	4	120	Lateral neck dissection^a^	N/A	N/A	None	N/A
Lombardi et al (2007, 30)	Case series/ neck	2	60 (LND only)	II-IV, Vb	25(21-29)	N/A	Transient hypocalcemia (2) Thoracic duct leak (1)	N/A none
Miccoli et al (2008, 31)	Case series/ neck	2	60 (LND only)	II-IV	8.5 (8–9)	N/A	None	6 none
Wu et al (2013, 32)	Case series/ neck	26	46 (LND only)	IIa, III-IV	8.3(3-21)	3.6(2-8)	Transient hypocalcemia (4) Transient RLN palsy (2)	19 none
Zhang et al (2014, 33)	Case series/ neck	26	210	II-V (20 cases) II-IV (6 cases)	38.6(31-50)	N/A	Spinal accessory nerve injury (1)	N/A none
Li et al (2016, 34)	Comparative/ neck	28	220.8	IIa, III-IV, Vb	30.4	3.4	Chyle leak (2)^b^ Skin paresthesia (8)	14-32^c^ none
Zhang et al (2017, 35)	Comparative/ neck	54	198.8	II-IV, Vb	41.1	4.8	Transient hypocalcemia (9) Transient RLN palsy (4) Hematoma (1) Wound infection (1) Horner’s syndrome (1) Chyle leak (1)	18.6 none
Zhang et al. (2017, 36)	Comparative/ neck	32	176	II-IV, Vb	44.6	4.8	Transient hypocalcemia (4) Transient RLN palsy (1) Seroma (1) Horner’s syndrome (1) Chyle leak (1)	>12 none
Xu et al (2020, 37)	Comparative/ neck	30	210.5	IIa, III-IV, Vb	31.4	6.73	Transient hypocalcemia (5) Transient RLN palsy (1) Accessory nerve injury (2) Chyle leak (1) Skin paresthesia (3)	6-21^c^ N/A
Ma et al. (2022, 38)	Comparative/ neck	34	172.6	II-IV	27(22-36.5)	6(4-7)	Transient hypocalcemia (11) Transient RLN palsy (5)	N/A N/A

a. Did not specify level or describe procedure.

b. Authors did not report if additional complications occurred.

c. Range in months, authors did not provide a mean follow up. NA, not applicable.

**Table 2:** Video-Assisted Anterior Chest Approach.

Author (Year of publication; citation number)	Study Design/ Approach	No. of Cases	Mean Operative Time (min)	Levels Dissected	Lymph Node Yield mean(range)	Mean Length of Stay in Days(range)	Complication (cases)	Mean Follow up (months) and recurrence
Kitagawa et al. (2003, 39)	Case series/ chest	3	264	Lateral neck dissection^a^	N/A	6.5(5-8)	None	N/A N/A
Lin et al. (2021, 40)	Comparative/ chest	31^b^	135	II-IV	18(16-21)	5(5-6)	Transient hypocalcemia (1)	48 none

a. Did not specify level or describe procedure.

b. 2 patients underwent bilateral LND and their data is not included. NA, not applicable.

**Table 3:** Anterior Chest/Breast Approach.

Author (Year of publication; citation number)	Study Design/ Approach	No. of Cases	Mean Operative Time (min)	Levels Dissected	Lymph Node Yield mean (range)	Mean Length of Stay (days)	Complication (cases)	Mean Follow up (months) and no. of recurrences
Li et al. (2011, 41)	Case series/ Endoscopic	11	94.3 (LND only)	II-IV	18.3 (9-26)	4.3	None	5.6 none
Yan et al. (2015, 42)	Case series/ Endoscopic	12	243	II-IV	21.8 (5-42)	5	Transient hypocalcemia (1) IJV injury (1)	N/A N/A
Guo et al. (2019, 43)	Comparative/ Endoscopic	18	235	II-IV	22.1	N/A	Transient hypocalcemia (4) Transient RLN palsy (1) Chyle leak (1) Large blood vessel injury (2)	N/A none
Wang et al. (2019, 44)	Case series/ Endoscopic	37	338.2	II-IV (22) II-IV, Vb (14) III-IV (1)	33.5 (5-56)	5	Transient hypocalcemia (12) Permanent hypocalcemia (1) Transient RLN palsy (3) Accessory nerve injury (1) Horner’s syndrome (1) Chyle leak (1) Chest wall ecchymosis (1) Skin burn (1)	24 1
Yan et al. (2021, 45)	Comparative/ Endoscopic	155	278.2	II-IV	22.91	6.02	Transient RLN palsy (8) Hematoma (3) Wound infection (2) Chyle leak (4) IJV rupture (19) Limb lift restriction (6)	N/A 2
Chen et al. (2022, 46)	Case series/Endoscopic	35	307.5	II-IV	24.2	5.9	Chyle leak (1) Cervical plexus injury (7) Accessory nerve injury (3) Hypoglossal nerve injury (1) IJV injury (2)	18.1 none

NA, not applicable.

**Table 4:** Transoral Vestibular Approach.

Author (Year of publication; citation number)	Study Design/ Approach	No. of Cases	Mean Operative Time (min)	Levels Dissected	Lymph Node Yield mean (range)	Length of Stay (days)	Complication (cases)	Mean Follow up (months) and no. of recurrences
Tan et al. (2020, 24)	Case series/Endoscopic	20	146	III-IV	10.9(6-16)	6.8	Transient RLN palsy (1) Seroma (2)	24.3 none
Tae et al. (2020, 47)	Case report/ Robotic	1	55 (LND only)	II-IV	29	N/A	None	N/A
Ngo et al. (2021, 48)	Case report/ Endoscopic	1	170	II-IV	8	N/A	None	N/A
Tae et al. (2022, 49)	Case series/ Robotic	14^a^	299^b^	III-IV (9) II-IV (1) II-V (2) III-V (1)	30.7	N/A	Transient hypocalcemia (3) Transient RLN palsy (1) Chyle leak (1) Seroma (4)	14.5 none

a. 10 patients had levels III-IV resected via transoral approach alone, 4 patients also had a retroauricular incision to get levels II-V.

b. For transoral alone. The combined cases were mean operative time of 431min. NA, not applicable.

**Table 5:** Anterior Breast and Transoral Approach.

Author (Year of publication; citation number)	Study Design/ Approach	No. of Cases	Mean Operative Time (min)	Levels Dissected	Lymph Node Yield mean (range)	Mean Length of Stay (days)	Complication (cases)	Mean Follow up (months) and no. of recurrences
Kuang et al. (2022, 50)	Case series/ Endoscopic	13	362.1	II-IV	36.6	N/A	Transient hypocalcemia (2) Transient chin numbness (3)	59 none
Chen et al. (2022, 18)	Case series/ Endoscopic	24	298.1	II-IV	27.9	5.8	Transient hypocalcemia (10) Transient RLN palsy (1) Chyle leak (1) IJV injury (1)	7.9 none
Wang et al. (2023, 51)	Case report/ Endoscopic	1	437	Lateral neck dissection^a^	66	N/A	Transient chin numbness (1)	9 none
Wang et al. (2023, 52)	Comparative/ Endoscopic	12^b^ 13^c^	256.0^b^ 336.9^c^	II-IV^b^ II-IV^c^	32.3^b^ 271^c^	4.9^b^ 5.0^c^	Transient hypocalcemia (2^b^, 3^c^) Transient RLN palsy (1^b^, 1^c^) Accessory nerve injury (1^b^) Horner’s syndrome (1^c^)	N/A N/A

a. Did not specify levels or describe procedure; IJV internal jugular vein.

b. Results for group that underwent 7-step LND via breast and transoral approach.

c. Results for contrast group that underwent standard LND via breast and transoral approach. NA, not applicable.

**Table 6:** Transaxillary Approach.

Author (Year of publication; citation number)	Study Design/ Approach	No. of Cases	Mean Operative Time (min)	Levels Dissected	Lymph Node Yield mean (range)	Mean Length of Stay (days)	Complication (cases)	Mean Follow up (months) and no. of recurrences
Kang et al (2009, 53)	Case series/ Robotic	13	286.9	II-Vb^a^	18.8 (7-28)	5.3	N/A	N/A
Kang et al. (2010, 54)	Case series/ Robotic	33^b^	280.8	IIa, III-IV, Vb	33	5.4	Transient hypocalcemia (17) Transient RLN palsy (2) Seroma (4) Chyle leak (3)	14 none
Kang et al. (2012, 55)	Comparative/ Robotic	56	277.4	IIa, III-IV, Vb	31.1	3.92	Transient hypocalcemia (27) Transient RLN palsy (2) Seroma (5) Hematoma (1) Chyle leak (5)	>12^c^ none
Yoon et al (2013, 56)	Comparative/ Robotic	73	297.9	II-IV, Vb	31.3	6.1	Transient hypocalcemia (35) Transient RLN palsy (3) Seroma (7) Hematoma (1) Chyle leak (4)	12 none
Lee et al. (2013, 57)	Comparative/ Robotic	62	271.8	IIa, III-IV, Vb	32.8	6.9	Transient hypocalcemia (24) Transient RLN palsy (2) Wound issue (2) Chyle leak (1)	8.4 none
Song et al. (2015, 58)	Case series/ Robotic	11^e^	298.2	II-V	19.82	9.18	Transient hypocalcemia (5) Chyle leak (1)	38 1
Song et al. (2016, 59)	Comparative/ Robotic	25^f^	298	II-V	27.3	8	Transient hypocalcemia (11) Transient RLN palsy (1) Seroma (1) Chyle leak (2)	29 1
Garstka et al (2018, 60)	Comparative/ Robotic	4	334.5	Lateral neck dissection^g^	16	1.8	Chyle leak (1)	7 none
Kim et al. (2018, 61)	Case series/ Robotic	334	286	I-V	34.1	N/A	N/A	N/A N/A
Kim et al. (2022, 62)	Case series/ Robotic	500	293.71	IIa, III-V	36.02 (8-46)	5.47	Transient hypocalcemia (152) Permanent hypocalcemia (20) Transient RLN palsy (20) Permanent RLN injury (5) Seroma (16) Hematoma (3) Chyle leak (26) Horner’s syndrome (2) Vagus nerve injury (1) Wound infection (1)	N/A 5

a. 11 patients underwent II-Vb dissection, 2 underwent selective lateral neck dissection and levels were not specified.

b. With an additional 0.8cm incision on the medial chest.

c. Follow up at 12 month for 36 patients.

d. With additional incision on ipsilateral breast areola.

e. Mixture of transaxillary alone and transaxillary with an additional small incision on the ipsilateral border of breast areola, numbers of each not specified.

f. 16 cases were via gasless transaxillary with additional 0.5cm incision 2cm from main transaxillary incision and 9 cases had additional 0.8cm incision on ipsilateral border of breast areola.

g. Did not specify levels or describe procedure. NA, not applicable.

**Table 7:** Bilateral Axillo-Breast Approach.

Author (Year of publication; citation number)	Study Design/ Approach	No. of Cases	Mean Operative Time (min)	Levels Dissected	Lymph Node Yield mean(range)	Mean Length of Stay (days)	Complication (cases)	Mean Follow up (months) and recurrence
Seup Kim et al. (2015, 63)	Comparative/ Robotic	13	382.3	II-IV, Vb	28.9	5.4	Chyle leak (1)	15.9 none
Choi et al. (2017, 64)	Case series/ Robotic	28	272.7^a^	IIa, III-IV, Vb	20.7^a^	3.1^a^	Transient hypocalcemia (7) Transient RLN palsy (1) Chyle leak (1) Horner’s syndrome (1)	17.3 none
Yu et al (2018, 65)	Case series/ Robotic	15	272.7	II-V	20.7	3.1	Transient hypocalcemia (7) Transient RLN palsy (1) Horner’s syndrome (1)	18.7 none
Paek et al (2020, 66)	Comparative/ Robotic	28	382.3	II-IV	36.5(31-50)	4.5	Transient hypocalcemia (2) Transient RLN palsy (3) Permanent RLN palsy (1) Chyle leak (1)	N/A N/A
Song et al. (2020, 67)	Case series/ Robotic	4	533^b^	I-V	54.5^c^	6.5	Pleural effusion (1)	27.3 none
He et al (2020, 68)	Case series/ Robotic	260	201	II-IV, Vb	17.9(7-41)	6.5	Transient hypocalcemia (51) Transient RLN palsy (3) Seroma (3) Tracheal fistula (1) Wound infection (1) Chyle leak (2)	28.6 1
Choi et al (2021, 69)	Comparative/ Robotic	12	277.08	IIa, III-IV, Vb	21.17	3.92	Transient hypocalcemia (2) Transient RLN palsy (1)	N/A none

a. n=15.

b. All bilateral LND.

c. Includes level VI. NA, not applicable.

**Table 8:** Retroauricular Approach.

Author (Year of publication; citation number)	Study Design/ Approach	No. of Cases	Mean Operative Time (min)	Levels Dissected	Lymph Node Yield mean(range)	Mean Length of Stay (days)	Complication (cases)	Mean Follow up (months) and recurrence
Byeon et al. (2014, 70)	Case series/ Robotic	4	306.1	II-V	33.1	11	Transient hypocalcemia (2) Seroma (1) Chyle leak (1)	11.3 none
Lira et al. (201, 71)	Case series/ Robotic	15^a^	340^b^	II-V^c^	27.2(17-40)^d^	3.4^b^	Transient hypocalcemia (2) Transient RLN palsy (3) Wound infection (1) Chyle leak (1)	17.4 none

a. Eight cases had additional mini-Kocher incision.

b. For the 12 total thyroidectomy +LND cases.

c. Level II-III under direct vision and IV-V via robot.

d. Includes VI.

**Table 9:** Transaxillary and Retroauricular Approach.

Author (Year of publication; citation number)	Study Design/ Approach	No. of Cases	Mean Operative Time (min)	Levels Dissected	Lymph Node Yield mean(range)	Mean Length of Stay (days)	Complication (cases)	Mean Follow up (months) and recurrence
Byeon et al. (2012, 72)	Case report/ Robotic	1	142	III-IV	7	8	None	N/A none
Kim et al. (2014, 73)	Comparative/ Robotic	22	209.4	II-V	33.14	9.2	Transient hypocalcemia (6) Transient RLN palsy (2) Hematoma (1) Seroma (2) Chyle leak (1) Ear lobe numbness (6)	15.9 none

NA, not applicable.

## 3 Video-assisted lateral neck

### 3.1 Anterior neck approach

**Figure 1:**
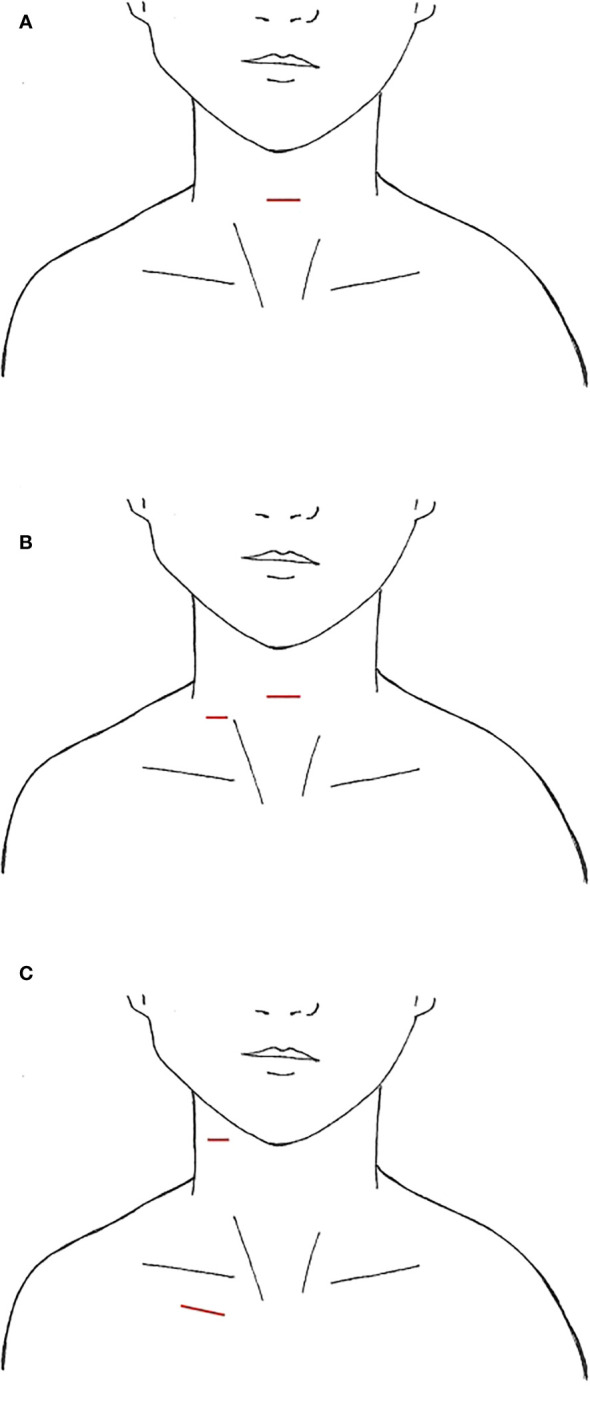
Video-Assisted Approaches, incisions marked in red **(A)** anterior neck **(B)** with lateral small neck incision **(C)** anterior chest +/- lateral neck.

First pioneered for benign thyroid nodules, the minimally invasive video-assisted thyroidectomy was performed by Miccoli et al. in 1998, with the expansion of the approach for papillary thyroid cancer and subsequently video-assisted SLND (VASLND) (25, 26, 30–33). There were 10 studies (**Table 1**) that described this technique, performed through a 2-5cm transverse incision in the anterior neck between the cricoid cartilage and the sternal notch, the conventional incision for a transcervical thyroidectomy (**Figure 1A**). Thyroidectomy and CND are performed transcervical and then endoscopic instruments and endoscope are placed through the incision to perform LND without extension of the incision. This is done without insufflation, with various retractors or retracting systems to externally displace the skin flap for operative exposure (30, 35). In Miccoli et al, a modified technique was performed, with thyroidectomy done through a smaller 1.5cm incision above the sternal notch and the VASLND performed via a lateral video-assisted approach requiring a second 5-7mm incision along the posterior border of the SCM (**Figure 1B**) (31). In Li et al, their LND is performed by placing 5mm trocar just superior to the clavicular head to accommodate blunt dissecting forceps while the laparoscope and other working instruments are placed through the 4-5cm anterior neck incision (34). After the working space is created, an additional 2mm retractor is then placed through the lateral neck skin ipsilateral to the LND to retract the jugular vein (34). In all studies, the transverse cervical artery, phrenic nerve, and spinal accessory nerves were identified and preserved.

#### 3.1.1 Indications and contraindications

The indications for video-assisted thyroidectomy with SLND generally included unilateral PTC with a primary tumor <4cm, no extrathyroidal extension, LNM <2 cm, no prior neck surgery or radiation, and no evidence of thyroiditis. Contraindications included distant metastases, LNM at level I or Va, or preoperative invasion of major neurovascular structures, trachea, or esophagus (32, 35). Xu et al. also excluded patients who had previous neck surgery or radiation and bilateral cervical LNM (37).

#### 3.1.2 Outcomes and complications

Among studies, the mean LN yield was 25-41, except in two studies that had a much lower yield of 8.3 (range 3-21) and 8.5 (range 8-9) (30–38). Mean operative time for VASLND alone was 46-60 minutes, but when included with thyroidectomy and CND, mean operative times were 198-220 minutes (30–34, 37). In Zhang et al, hospital length of stay (LOS) were similar between groups but the operative times were longer in the VASLND, 198.8±19.6 min vs 172.3±28.0 min (35). In 2 other studies, LOS was shorter in the VASLND by 2 days (33, 34).

Among studies with at least 12 months of follow-up, there were no reported recurrences, sTg levels were low or undetectable, and there were no differences in postoperative complications between groups, including transient hypocalcemia or hoarseness, Horner’s, seroma, hematoma, wound infections, or chyle leak (32–36). There were no cases of permanent RLN injury or hypocalcemia in the studies. Zhang et al’s group reported one serious complication comprising an injury to the trapezius branch of the spinal accessory nerve that resolved after 18 months (33).

All patients in the VASLND groups were satisfied with their cosmetic results (32, 34, 35). In a study by Zhang et al, the cosmetic outcomes were evaluated by a numerical and verbal response scales, evaluated in both the VASLND group (n=54) and transcervical group (n=38) at 1 year after surgery. Both scale scores were better in the minimally invasive group, which was statistically significant (35). In another study by Zhang et al, these results remained significant (33).

#### 3.1.3 Advantages and limitations

The biggest advantage to this approach is avoidance of a long collar or L-shaped incision with a better cosmetic outcome and less tissue trauma. However, the tradeoff is longer operative times. Despite longer anesthesia, this approach leads to a quicker recovery time with comparable oncologic outcome (33). Zhang et al. argues that dissecting level II, particularly IIb, is easier endoscopically than transcervical; however, the procedure requires around 40 cases to become facile (33). In contrast, level I and V can be challenging to resect (32). An additional advantage to this approach is that it doesn’t require special instrumentation - although the use of plastic surgery or otolaryngology instruments may occur and in China, the use of special working space retractors and suction retractor has been described (35).

### 3.2 Anterior chest/infraclavicular and lateral neck approach

LND via the gasless anterior chest or infraclavicular approach (ACA) was first described by Shimizu et al, and again reported in two additional studies (39, 40, 74). This technique entails the use of a 3.5-5cm ipsilateral incision 3-5cm inferior to the clavicle (**Figure 1C**), through which the harmonic scalpel and grasper are placed. Thyroidectomy was performed through this incision. For some patients, an additional 0.5cm incision was made on the ipsilateral lateral neck, through which the endoscope was placed, and this was enlarged to 2-2.5cm for LND and accommodation of the harmonic scalpel and grasper (39). Subplatysmal dissection is performed under direct vision from the infraclavicular incision, the SCM is divided longitudinally between its two heads, and a working space underneath the strap muscles is developed (40). Then either an external retractor is used, or Kirchner wires are inserted through the subcutaneous tissue transversely to tent the skin up and create a working space (39, 40). In the small case series by Kitagawa et al, 8 patients with PTC underwent either hemi- or total thyroidectomy with LND by ACA over a 4-year period (39). In Lin et al, 91 patients underwent total thyroidectomy, CND, and SLND (level II-IV) over a10-year period, of which 31 were in the ACA group and 60 were in the transcervical group (40). Only 5 patients in this series required the lateral neck incision, while the rest of the cases were completed entirely through the anterior chest incision. One to two drains were placed in the surgical bed and removed prior to discharge.

#### 3.2.1 Indications and contraindications

In Lin et al, inclusion criteria for thyroidectomy, CND, and LND using a video-assisted anterior chest approach were well differentiated PTC, tumors <3cm, lack of extrathyroidal extension, suspected clinically positive level II-IV LNM with LN <2cm in size (40). Kitagawa et al. was more stringent on a primary tumor size <1cm, although two patients in their sample were between 1-2cm (39). Patients with suspected level I or V LNM were excluded, as were those with a prior neck surgery or radiation, suspected perinodal infiltration of LNM, or distant metastases (40).

#### 3.2.2 Outcomes and complications

**Table 2** summarizes the 2 studies on this approach. In Lin et al, the only significant difference in outcomes between groups was a longer operative time in the ACA group (mean 135 min vs 108 in transcervical group) and lower intraoperative blood loss (60 ml vs 100 ml in transcervical group) (40). Only Lin et al. noted the LN yield, which was 16-21 and similar to the transcervical group. Mean follow up time was 48 months for the ACA group and 35 months for the transcervical group. There was no remnant thyroid tissue on imaging, mean serum thyroglobulin (sTg) levels were <1ng/mL at 6 months postoperatively in both groups, and there was no recurrence in the follow up periods. Importantly, the functional outcomes (voice swallowing, arm abduction, and neck impairment) were not different between groups and cosmetic assessment scores demonstrated ACA patients were more satisfied with their results (40). The authors also noted that while the operative time was longer for the ACA group, this decreased over time as they became more facile at the procedure. The only post operative complications were transient hypoparathyroidism, 1 case in the ACA group and 2 in the transcervical group, all of which resolved within 2 weeks. In Kitagawa’s cohort, there was one intraoperative anterior jugular vein injury during the subplatysmal dissection (39). There were no RLN injuries or post operative hemorrhage in either study.

#### 3.2.3 Advantages and limitations

The advantages of this approach are a larger working space and elimination of an anterior neck scar compared to the ANA and less flap dissection than remote access techniques (such as transaxillary or breast). Further, total thyroidectomy is possible via this approach. With the combination of the lateral neck incision, level II can be synergistically dissected with the anterior chest incision, whereas in the transoral approach, level II is very difficult to access. An additional advantage to this gasless technique is no CO^2^ insufflation and potential complications that accompany it. Limitations to this approach include difficulty with dissection of the contralateral LN in the tracheoesophageal groove and a relatively large anterior chest scar (40).

## 4 Endoscopic remote access approaches

### 4.1 Anterior chest/breast approach

**Figure 2:**
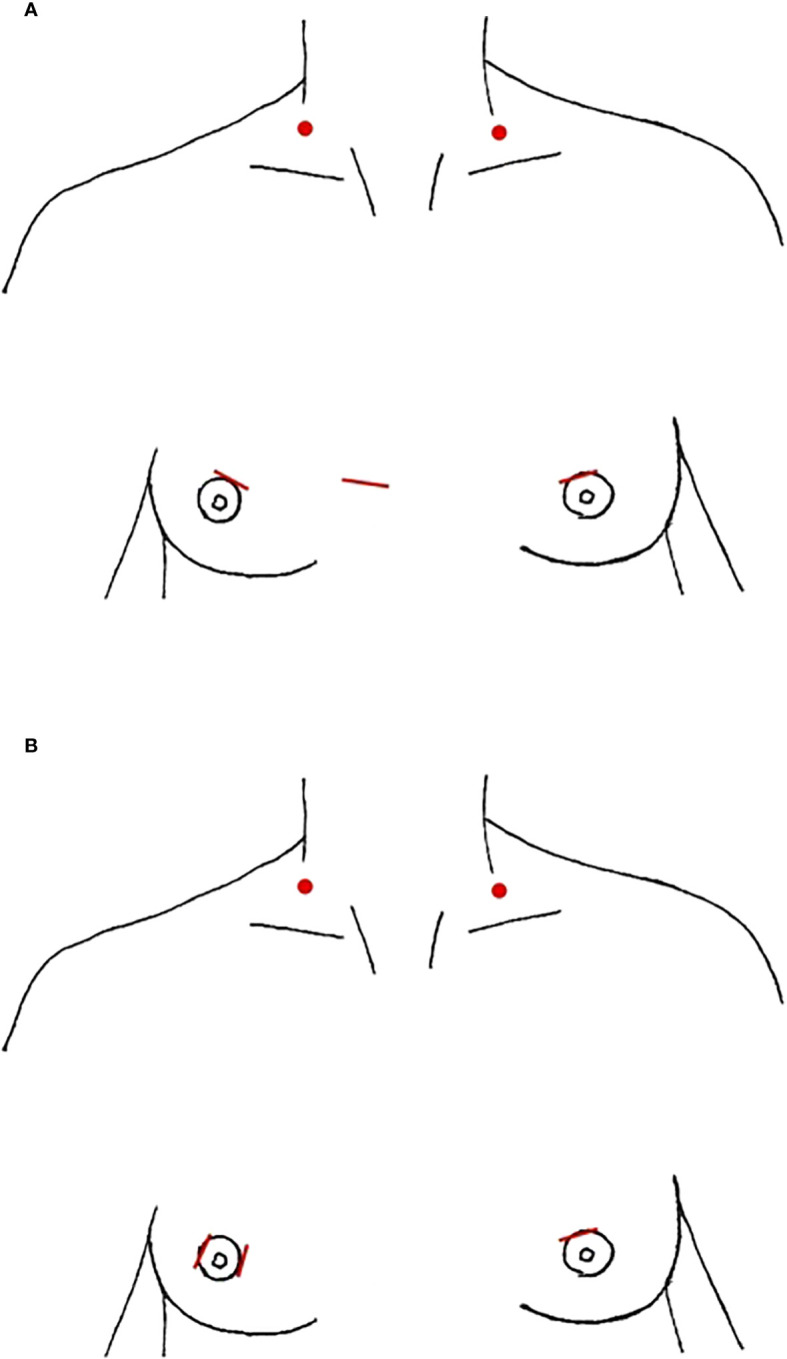
Anterior Breast Approach, incision location marked in red, red dots denote location of needle-assisted retractor placement **(A)** parasternal **(B)** periareolar only.

The anterior chest/breast approach (ABA) is the most commonly performed purely endoscopic LND approach. We identified 6 studies that detailed this technique in 300 patients, all from South Korea or China (**Table 3**). The majority entailed a 1-1.2 cm parasternal incision at the nipple line for a 10 mm trocar with a peri-areolar incision on each breast to accommodate 5 mm trocars, although Guo et al. describe an additional peri-areolar incision rather than parasternal (**Figure 2**) (43–45). The subcutaneous working space in the chest is created with an epinephrine solution or a liquid-gas tumescent solution and blunt dissection, and is maintained with CO_2_ insufflation at 6mmHg (41, 44, 45). The working space in the neck is further developed between the platysma and the strap muscles using blunt dissection and the ultrasonic scalpel up to the thyroid cartilage superiorly and laterally to the SCM. The thyroidectomy and CND occur similar to a conventional transcervical approach. Then the lateral working space is created to the lateral edge of the SCM and the superior border of the digastric posterior muscle belly. This dissection is followed by division of the SCM longitudinally between the two heads up to the carotid bifurcation, as well as dissection between the anterior SCM and the strap muscles. Some studies report division of the omohyoid muscle while others preserve it (46, 50). In multiple studies, needle-assisted instruments were also used to maintain the working space in the neck, including two U shaped retractors inserted at the inferior aspect of the SCM bilaterally, separating the two SCM heads (41, 42, 44). In Wang et al. they also utilize a 3mm grasper inserted at the midclavicular line and second rib intersection, and skin suspension suture on the anterior neck requiring 4 surgeons to complete the procedure (44). Levels II-IV were routinely resected in all studies, with the addition of Vb in Wang et al. (44). The dissection occurs in a similar manner to a transcervical procedure, taking care to identify and protect the cervical plexus, phrenic nerve, vagus nerve, and the transverse cervical artery, while skeletonizing the spinal accessory nerve and IJV in level II (18, 41, 43–45). Two drains were typically left in the thyroid fossa, one centrally and one laterally, both of which were removed prior to discharge from the hospital.

#### 4.1.1 Indications and contraindications

Inclusion criteria varied slightly, but the indications for the anterior chest/breast approach generally included patients with differentiated thyroid cancer (DTC) with a primary tumor less than between 2-4cm (depending on the study), suspected or confirmed lateral LNM, LNM diameter <2cm, and a cosmetic demand. Contraindications were previous neck surgery or radiation, distant metastases, level I or V disease (except in the studies that included Vb), fixed or fused LNM, or surrounding tissue invasion (i.e., trachea, esophagus, IJV, RLN). In Wang et al, the authors excluded recurrent disease. Guo et al. excluded patients >60 years-old and Yan et al. >50 years-old (42–44).

#### 4.1.2 Outcomes and complications

Over all studies, mean operative times for endoscopic subtotal and total thyroidectomy, CND, and LND ranged from 235 min to 338.2 min (**Table 3**). These were significantly longer than transcervical groups by a mean difference of 53-99 min (43, 45). In two studies that reported it, LOS was similar across groups (43, 45). Mean LN yield ranged from 18.3 to 35.5, the upper end of the range likely due to the addition of level Vb.

Only 3 studies noted the mean follow up time, ranging from 5.6 to 24 months. Serum thyroglobulin (sTg) was only noted in one study and was low in the majority of patients (44). Three patients (0.97%) had local recurrence (44, 45) (75). There was no statistical difference in the LN yield between groups or post operative complications. In the studies that compared operative times, they found the EALND group to be longer but they had shorter length of hospital stay (LOS) (43, 45). Further, the EALND group had less neck discomfort and better cosmetic scores, like other studies (43, 75).

Among the comparative studies, there were no other significant differences in complications rates between the ABA and transcervical groups, with the exception of Yan et al’s rate of IJV rupture (43, 45). They found IJV rupture was significantly higher in the ABA group than transcervical, 12.26% (n=19/155) vs 2.94% (n=3/102), p<0.01 (45). The authors hypothesized multiple reasons for the increased rate of IJV injury, including accidental clamping of the vessel during dissection, more wall tearing due to no direct touch with the vessels, and limited working space and noted that all were repaired endoscopically with no conversions to open in their 155 cases (45). Across studies, there were 268 patients and only 1 developed permanent hypoparathyroidism (0.37%), 17 temporary hypocalcemia (6.34%), 12 temporary RLN palsy (4.48%), 4 accessory nerve injuries (1.49%), 2 wound infections (0.75%), 24 IJV or large blood vessel injury (8.96%), 7 chyle leaks (2.61%), 1 Horner’s syndrome (0.37%), 1 chest wall ecchymosis (0.37%), and 1 skin burn (0.37%) (41–46). One hypoglossal nerve injury (0.37%) and 7 cervical plexus injuries (2.61%) were additionally reported, all in the same study (46).

#### 4.1.3 Advantages and limitations

The major advantage of this approach is the improved cosmetic results compared to transcervical or video-assisted approaches as this technique circumvents an anterior neck scar. It is less expensive than robotic approaches (43, 45). Similar to the video-assisted infraclavicular approach, Wang et al. state that dissection of level IIb was easier via ABA compared to the transcervical approach, and the symmetric view makes a total thyroidectomy easier than a unilateral approach, such as transaxillary or retroauricular (44). Disadvantages include a steep learning curve, the need for careful patient selection, expert knowledge of the anatomy to avoid complications, and a longer operative time compared to the conventional approach (43, 45). There may also be an inadvertent blind spot due to the clavicle, making level IV (and VI) dissection difficult via this approach. Furthermore, if there are suspected LNM in level I or V, authors note that necessitates a different approach or the addition of a neck incision (43). One concern about endoscopic techniques that utilize CO_2_ insufflation is the potential for tumor rupture and spillage via the “chimney effect” (41, 76). Kim et al. published a case report of tumor recurrence in the subcutaneous tunnel and operative bed after ABA but did note the presence of a capsular tear intraoperatively (76). This was the only report of track recurrence that was identified, but it should be kept in mind in the event of intraoperative tumor rupture. Lastly, while the LOS did not differ between groups in these studies, that may be less generalizable in countries where the LOS after transcervical LND are shorter than in Asian countries and different health systems (69).

### 4.2 Transoral vestibular approach

**Figure 3:**
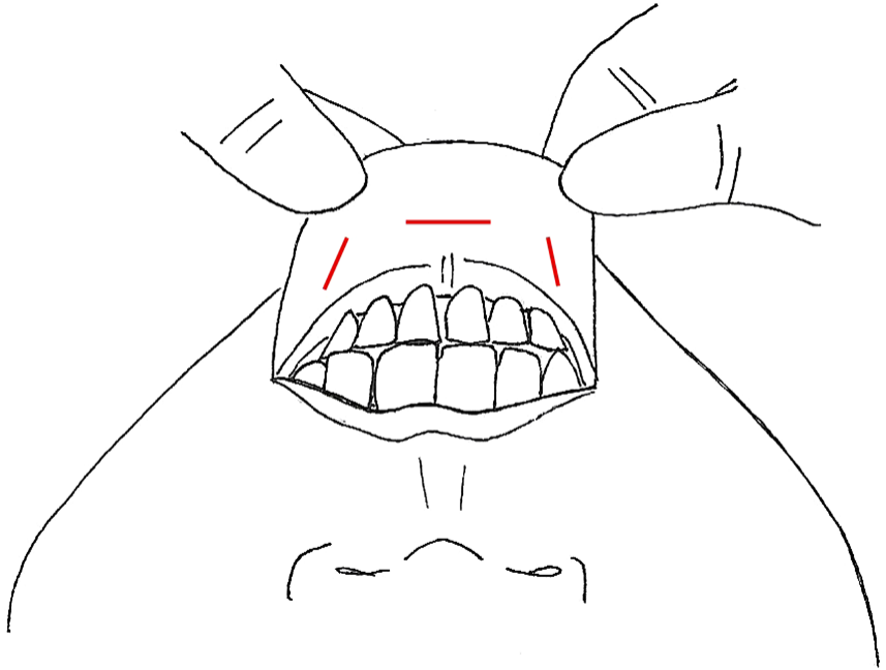
Transoral Approach, incision location marked in red.

The transoral endoscopic thyroidectomy vestibular approach (TOETVA) has become more popular in the last few years and has even gained traction in North America (19, 21). This technique has been expanded to include LND in only a small case series and a case report (**Table 4**) (24, 48). TOETVA is performed through a three-port technique in the oral vestibule, including a midline 10mm port for the laparoscope and 2 additional 5mm working ports laterally (**Figure 3**) (77). The subplatysmal space is developed through an epinephrine solution hydrodissection and blunt dissection with dilators down to the sternal notch inferiorly and to the SCM bilaterally. After trocars are inserted, insufflation is kept low at 6mmHg. Thyroidectomy and CND proceed via a top-down approach. In Tan et al’s case series of 20 patients, they also performed dissection of level II-IV through these ports; however, in Ngo et al, they describe adding an additional 5mm trocar near the right 6^th^ teeth to complete their right sided dissection of level II (24, 48). Similar to other remote access techniques, the SCM is split longitudinally and the LND proceeds through that space, with the resection of the omohyoid to open up the working space (24). In their case series, Tan et al. left a small 3mm drain in the operative bed, exiting through a small skin puncture on the chin. The drains were removed prior to discharge.

#### 4.2.1 Indications and contraindications

Indications for endoscopic transoral thyroidectomy and LND were unilateral PTC with primary tumor <2cm. Tan et al. also limited their study to lobectomies (24). Additional inclusion criteria were no capsular or surrounding structure invasion, suspected/confirmed LNM in level III or IV only, no thyroiditis, and strong cosmetic demand. Contraindications were previous neck surgery or radiation, distant metastases, fused or fixed nodes, and BMI >30 (24). Higher BMI was a contraindication due to the technical challenges of creating skin flaps in higher BMI patients.

#### 4.2.2 Outcomes and complications

In Tan et al’s cohort, the mean LOS was 6.8 ± 1.8 days with a mean operative time of 146 ± 18.7 minutes compared to Ngo et al’s operative time of 170 minutes. The LN yield was relatively low in both studies, with 10.9 (range 6-16) for level III-IV and 8 LN in the case report despite Ngo et al’s addition of level II. Mean follow up was 24.3 months, and there was no residual disease or recurrences on follow up imaging (24). One patient (5.00%) developed transient RLN palsy, which recovered within a month and a half (24). Two patients (10.00%) developed seromas, with resolution after drainage at two weeks postoperative.

#### 4.2.3 Advantages and limitations

Advantages to this natural orifice approach include the avoidance of a neck scar. It also has a smaller dissection tract to the neck and remains minimally invasive compared to other remote access techniques that require a large dissection area, such as anterior chest/breast, axillary, and bilateral axillo-breast approaches. It also provides a symmetric view, allowing for total thyroidectomy and CND. Additionally, while this technique can be performed robotically, costs are lower with the endoscopic technique using laparoscopic instruments. When comparing the mean operative times of this approach to other remote access techniques, transoral are shorter, albeit with longer operative times compared to transcervical LND. A disadvantage is the challenge of dissecting level II completely, and level V is also difficult to access due to lateral border of SCM, which calls into question its oncologic safety (18, 69). There are also the risks of mental nerve injury, skin trauma/burns, and as with any laparoscopic surgery using insufflation, CO_2_ embolism associated with this approach (47). Lastly, one disadvantage to all transoral procedures is the potential to introduce oral bacteria into the neck, necessitating prophylactic antibiotics in many institutions (21).

### 4.3 Breast and transoral approach

To overcome the limitations of dissection of level II and IV via endoscopic ABA or transoral approach, some institutions in China have combined the two approaches into endoscopic LND via breast and transoral approach (BTOA). Two case series, one comparative study, and a case report were identified for a total 63 patients (**Table 5**). All cases began with thyroidectomy via the breast approach, but the technique used for CND varied between these studies, with 2 studies performing it through the breast approach, while 2 other studies preferred CND through the transoral approach (18, 50–52). Afterwards, LND was done via the breast approach, dissecting level II-IV as previously described above (18, 50, 52). Interestingly, in Wang et al’s comparative study they evaluated surgical outcomes between 12 patients who underwent total thyroidectomy, CND, and LND (level II-IV) via BTOA by their 7-step process to 13 patients who underwent the same procedures via BTOA but the “contrast” group was not a fixed procedure. These cases still followed the same operative steps as the 7-step group, however isolation of the SCM and IJV occurred after thyroidectomy, CND was done via breast approach first and then supplemental dissection completed via transoral approach, and in half the cases both 5mm and 10mm laparoscopes were used requiring switching between them (52). All 25 cases were performed by the same surgeon and both group’s results are presented separately in **Table 5**. In studies that detailed their surgical operation, further dissection of the inferior border of level IV, at the venous angle of the IJV and subclavian vein and along the surface of the subclavian vein to the lateral border of the SCM was done transorally (18, 50, 52). Two drains were typically left, one in the central region and one in the lateral compartment and were removed prior to discharge.

#### 4.3.1 Indications and contraindications

General indications for BTOA were PTC with no severe invasion, confirmed LNM with largest diameter of <2 cm, and a strong cosmetic demand. Primary tumor size cutoff varied but was <4 cm in two studies, while Wang et al. had a more stringent size limit of < 2cm. Contraindications included prior neck surgery or radiation, metastases to level I or V, fused or fixed LNM, invasion of surrounding tissue (i.e., trachea, esophagus, RLN), distant metastases, oral abscess, uncontrolled hyperthyroidism, or thyroiditis. Chen et al. also excluded patients with BMI >40kg/m^2^ (18).

#### 4.3.2 Outcomes and complications

Mean post operative LOS was specified in 2 studies and was 4.9-5.8 days for 49 patients (18, 52). Mean operative times ranged from 256.0 to 362.1, with an outlier of 437 minutes for the case report by Wang et al. (**Table 5**). Mean LN yield appears to be slightly higher by this approach than ABA alone and much higher than transoral alone, with a mean LN yield for level II-IV between 27.1-36.6 nodes. In the case report by Wang et al, they harvested 66 LN but do not specify levels nor describe their technique (44). There were no formal assessments of cosmetic outcomes in these studies, but the authors do note that all patients were satisfied with the cosmetic results in all cases (18, 50, 52).

Overall complications included transient hypocalcemia (n=17/63, 26.98%) and transient RLN palsy (n=2/63, 3.17%), but no cases of permanent RLN injury, hypocalcemia, hematoma, seroma, or wound infections. In Chen et al’s case series of 24 patients, there was one case of chyle leak and IJV injury (18). In Wang et al’s comparison study, there was one case of Horner’s syndrome and 1 case of accessory nerve injury in the 7-step group (52). Transient chin or mandibular numbness also occurred in 4/63 patients (6.35%) but generally resolved within 2 weeks (50, 51).

#### 4.3.3 Advantages and limitations

An advantage to this approach is the lack of an anterior neck scar, similar to both the ABA and transoral approaches. The additional level IV transoral dissection did yield more LN than the breast approach alone, which were positive in 3/13 patients (23.1%) (50). While there were longer operative times compared to open cases in the ABA comparative studies, the addition of further dissection transorally does not measurably increase mean operative times compared to the ABA alone (**Table 3**). Again, a disadvantage of the transoral approach is the potential to introduce oral flora into the neck, often requiring prophylactic antibiotics.

## 5 Robotic remote access approaches

### 5.1 Transaxillary approach

**Figure 4:**
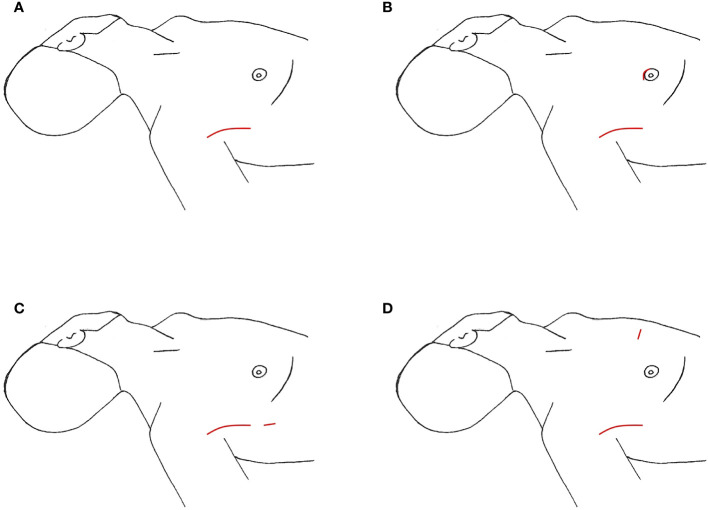
Transaxillary Approach, incision location marked in red **(A)** axillary incisions **(B)** additional incision on breast **(C)** additional incision on axilla **(D)** additional incision on chest.

Since its inception, initially endoscopically then robotically, thyroid surgery by gasless transaxillary approach (TAA) has gained popularity, first in Asia and then in North America (19, 21). LND via this approach was found in 9 South Korean studies and 1 North American study for a total of 1,111 cases, with the majority undergoing dissection of level IIa, III, IV, and Vb (**Table 6**) (53–59, 61, 62). The largest study was a case series of 500 patients over 11 years by Kim et al. (62). For this approach, the patient is supine with neck extended. The ipsilateral arm is abducted 80 degrees from the body or in an extended salute position with the arm flexed at 90 degrees (21, 54). A 4-8cm incision is created in the ipsilateral anterior axillary line behind the lateral border of the pectoralis major (**Figure 4**), intended to be obscured by the arm when in an anatomic position. A large subcutaneous flap is created under direct vision with electrocautery, traversing from the axilla above the pectoralis major, over the clavicle, and to the midline neck below the platysma. The working space is created superiorly to the posterior belly of the digastric muscle and submandibular gland and the anterior border of the trapezius laterally (2, 62). While creating this flap, care is taken to divide the external jugular vein at the SCM and identifying and preserving the spinal accessory nerve. Depending on the study, in order to completely expose the venous angle at the junction of the IJV and subclavian vein and complete the LND, the clavicular head of the SCM may be divided at the insertion point (54, 56). In Kim et al’s study, they dissected the lateral border of the strap muscles off the IJV to identify the thyroid, and the dissection continued between the anterior thyroid and strap muscles (62). A spatula shaped external retractor is placed through the incision to maintain the working space, retracting the strap muscles, SCM, and skin flap (54). In half the studies, all 4 arms of the robot are inserted through the axillary incision. In the remaining studies, 3 robotic arms were inserted into the axillary incision, and an additional 4^th^ trocar either through a 0.8cm peri-areolar incision, another anterior axillary 0.5cm incision 2cm inferior to the main axillary incision, or a 0.8cm incision on the anterior chest, approximately 2cm superior and 6-8cm medial to the ipsilateral nipple (**Figures 4B–D**) (53, 54, 58, 59). The robot is then docked, followed by total thyroidectomy, CND, and SLND of levels III, IV, Vb. To complete the level II dissection, the external retractor and robot required repositioning towards the ipsilateral submandibular gland (54, 56, 59, 62). A closed suction drain is left in the surgical bed, exiting the incision, and is removed prior to hospital discharge.

#### 5.1.1 Indications and contraindications

Indications for TAA LND generally included DTC with a primary tumor <4 cm, with minimal invasion to the anterior thyroid capsule and strap muscles, clinically positive small lateral LNM, and a strong patient preference/cosmetic demand. Contraindications were tumor invasion to surrounding structures (i.e., trachea, esophagus, RLN), multilevel LNM, perinodal invasion, prior neck surgery or radiation, a pathologic neck or shoulder condition, recurrent disease, or distant metastases (54–62).

#### 5.1.2 Outcomes and complications

Mean operative times for all studies ranged from 271.8 to 334.5 minutes (**Table 6**). In half the comparative studies, TAA was a significantly longer operation than an open transcervical thyroidectomy and LND, while the other studies found no significant difference in mean operative time (55–57, 60). Because larger body habitus has been suggested to make this approach more challenging, one large case series stratified operative times and complication rates by BMI less than or more than 25kg/m^2^ and found no significant difference between groups (62, 78). When they performed their sub-analysis with a BMI of 30kg/m^2^, there again were no differences between groups (62). Mean LN harvested via this approach ranged from 16-36.02, with >95% (1058/1111) patients having a mean LN yield of more than 30. Mean LOS was variable between South Korean studies, ranging 3.92 to 9.18 days. Kang et al. found that their robotic group had a significantly shorter hospital LOS compared to transcervical, 6.0 ± 2.5 vs 8.0 ± 5.2, p=0.008, respectively (55). In the North American study, mean LOS was 1.8 days (60). The longer length of stay across South Korean groups, compared to those in other countries, is attributed to health system differences, wherein South Korea medical insurance covers longer hospitalizations for cancer patients (57, 69). Two studies had a relatively short mean follow up times of 7 and 8.4 months, while the rest were 12 to 38 months.

Three studies had recurrences (58, 59, 62). In each of Song et al’s studies, there was one recurrence detected by ultrasound at level III, 18 months after surgery, despite no uptake on whole body iodine scans after RAI ablation and undetectable postoperative serum thyroglobulin (58, 59). Of the 5/500 recurrences in Kim et al’s series, one was in the contralateral neck, three were in the central neck, and one was in the central and contralateral neck (62).

In Lee et al, multiple quality of life parameters were also assessed, including neck and shoulder function (57). At 6 months after surgery, the transcervical group had a higher rate of swallowing impairment but showed no difference between groups in neck dissection impairment index, arm abduction test, or voice handicap index. The authors posited that this difference in swallowing impairment could be because the strap muscles are not divided at midline in TAA, perhaps resulting in less adhesions to the subcutaneous tissues. Questionnaires were used to assess pain at the surgical scar and cosmetic outcome scores. There were no differences in scar pain, but the cosmetic satisfaction for TAA was significantly higher due to the decreased visibility of scars (57). Similarly, Song et al. also assessed postoperative pain, paresthesia, and cosmetic satisfaction (59). They also found that the TAA group had higher anterior chest pain and paresthesia up to 1 month after surgery, but this difference was no longer significant by 3 months postoperatively. However, the higher cosmetic satisfaction scores of TAA remained significant throughout the follow up period.

Of the studies that reported complications (n=764), the most common complication was transient hypocalcemia, occurring in 271 patients (35.47%), followed by chyle leaks in 43cases (5.63%), 30 transient RLN palsies (3.93%), and 33 seromas (4.32%) (54–60, 62, 79). When looking at individual studies, the rates of transient hypocalcemia ranged from 30.40% to 51.52%. There were 20/500 cases (4.00%) of permanent hypocalcemia (defined as lasting more than 6 months) in the large case series by Kang et al, as well as 2 cases of Horner’s syndrome and 1 vagus nerve injury. Two studies had a total of 3 wound infections (57, 62).

#### 5.1.3 Advantages and limitations

Advantages to robotic surgery over endoscopic or a transcervical open procedure include better visualization, improved freedom of movement, and elimination of tremors. The available studies show comparable oncologic outcomes, with less swallowing symptoms, and the cosmetic satisfaction appears to be greater for TAA compared to a conventional approach, as discussed above (55, 57, 59). This approach being gasless, without the risk of complications related to CO_2_ insufflation is another advantage. Kim et al. also claim that there is decreased risk of traction injury to the RLN via this approach due to the oblique view (62).

A main concern regarding this technique is the risk of brachial plexus injuries from arm positioning. This can be mitigated by careful positioning, reducing traction on the brachial plexus by flexing the ipsilateral arm in a 90-degree position (21, 80). Further, injury to cervical plexus sensory nerves can lead to anterior chest paresthesia, which is commonly seen after TAA, although often temporary (21, 57). In Lee et al, the presence of paresthesia or hyperesthesia in the neck and anterior chest questionnaires demonstrated that changes in the anterior chest were significantly more common in the robotic group whereas the opposite was true in the neck, with the open group having a higher incidence of neck numbness (57). Additional concerns include possible injury to the thoracic outlet vessels, aerodigestive injuries, fibrosis, tract hemorrhage, tract recurrence, or adhesions to the IJV after thyroidectomy that make reoperation, by any approach, more difficult (80, 81). Due to the unilateral approach, if the patient requires a contralateral MRND, this is not feasible via this approach and would necessitate bilateral transaxillary incisions. Lastly, the increased operative time and costs associated with TAA may be prohibitive in many settings.

### 5.2 Bilateral axillo-breast approach

**Figure 5:**
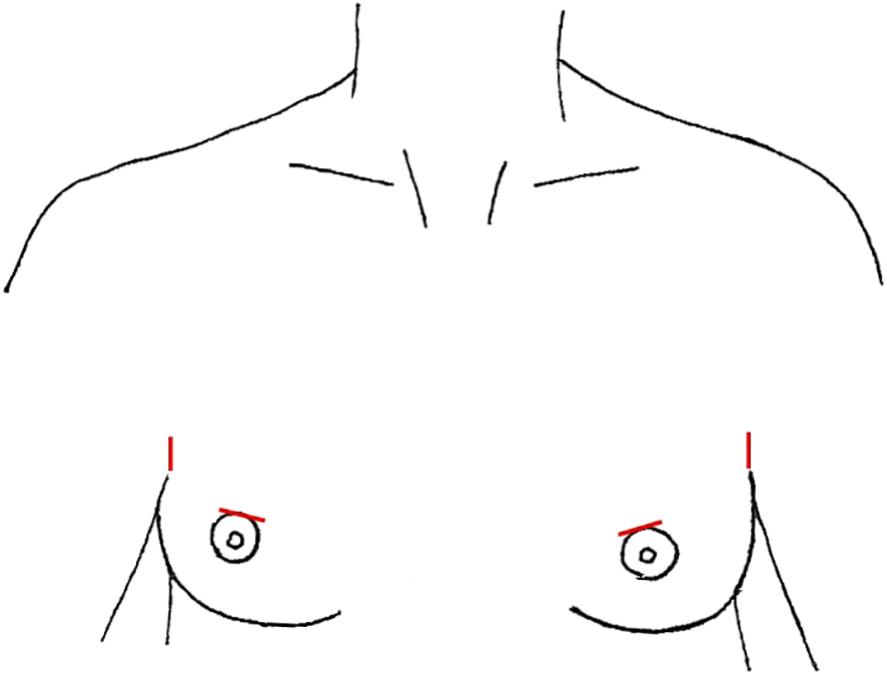
Bilateral Axillo-Breast Approach, incision location marked in red.

The bilateral axillo-breast approach (BABA) was first developed for thyroidectomy in South Korea in 2008 and has been expanded to MRND (82). The technique has gained popularity, predominantly in Asia where 7 studies have been published, the largest of which was a case series from China with 260 patients who underwent total thyroidectomy and LND of levels II-IV, Vb (**Table 7**) (63–69). Patient positioning is an important component of this technique. There is a pillow placed below the shoulders, arms are slightly abducted bilaterally, and the patient is placed in 25-30 degrees of reverse Trendelenburg (64). The circumareolar area is elevated by wrapping the lower portion of the breasts to raise the pivot point of the circumareolar trocars, obviating a blind spot in the lower neck and allowing better dissection (**Figure 5**) (63, 64, 83). Four incisions total are used: a 12mm upper areolar incision in the right breast and 8mm on the left, and vertical 8mm incisions in the axilla bilaterally. In male patients, the circumareolar incisions are moved slightly superior, 7cm below the clavicle (63). Hydrodissection is used on the anterior chest to aid in tract creation in one study (65). The tracts are then created with blunt dissection using a vascular tunneled, the robot is docked, and the working space creation is completed by ultrasonic scalpel. Insufflation is kept low, at 5-6 mmHg. For the LND, the boundaries of the working space superiorly are the submandibular gland and posterior belly of the digastric muscle, to the anterior border of the trapezius laterally, and 2cm below the clavicle inferiorly. After total thyroidectomy and CND, MRND is performed, either levels I-V or II-V depending on the study. The camera port is directed to the SCM and the camera is rotated towards side of MRND, clockwise for patient’s left and counter clockwise for right. The SCM is dissected from the sternohyoid, identifying the omohyoid and dividing the inferior belly to open the working space and see the IJV. Some studies split the SCM longitudinally to aid in dissection (68). Once the medial border of SCM is free, a transcutaneous suture is used to retract it laterally (63). Dissection of levels IV and Vb followed by level III and IIa was performed across all but one study, using conventional technique (63, 64, 68). Level I, Iib, and Va were included if there were suspicious nodes or clinical LNM in those levels. Like the transoral approach, the camera and robot arms may need to be repositioned prior to level II dissection for better visualization. After completion of LND, a drain is left through the ipsilateral axillary incision, which is removed prior to discharge home.

#### 5.2.1 Indications and contraindications

Indications for BABA included DTC with clinically suspicious or confirmed lateral LNM, and a cosmetic demand for the remote access technique. Contraindications generally included primary tumors >4cm, invasion into adjacent organs (i.e., trachea, esophagus, RLN), prior history of neck radiation or surgery, extranodal invasion, LNM that were fused or fixed or encased the common carotid or IJV, if the LNM were above the digastric muscle or below the clavicle, or distant metastases (63–65, 67–69).

#### 5.2.2 Outcomes and complications

Hospital LOS was 3.1 days to 6.5 days across studies (**Table 7**). One study found shorter LOS in the BABA group (3.92 vs. 4.71 days, p=0.056) (69). Mean operative times were significantly longer in the BABA group (201-382.3 minutes), but comparable to other remote access techniques. The outlier in the group, with a mean operative time of 533 minutes, consisted of four cases, all of which were bilateral LND (67). Interestingly, when Choi et al. subdivided their mean operative time to exclude flap creation, they found the operative times for the actual procedure were comparable to a transcervical approach, 200.33 ± 26.86 vs 191.43 ± 60.43 minutes, p=0.523 (69). He et al. note that all 260 patients had sensory impairment of the anterior chest, nipples, or neck, but this resolved within 4-12 months (68). All patients were either satisfied or extremely satisfied with their cosmetic results (68). The mean LN yield was 17.9 to 36.5 across studies, save for one that included level VI in their totals. There were no differences in LN harvest between BABA and transcervical groups (66, 69). Stimulated sTG levels were similar after RAI ablation (63, 69). Mean follow up times were 15.9 to 27.3 months, and there was one (0.30%) recurrence at level IV, for which the patient had an open procedure to remove one metastatic LN (68).

Across studies, there were 360 patients who underwent BABA, with 19.17% (n=69) developing transient hypocalcemia (range 7.14-46.67%). There were fewer cases of transient hypocalcemia in the BABA group than open in one comparison study (16.7% vs 53.6% transcervical, p=0.041), but no differences in other complication rates (69). The second most common complication was transient RLN palsy (9/360, 2.50%), with 1 case of permanent RLN palsy (66). Four studies had a total of 5 chyle leaks (1.39%), all managed conservatively, and two studies had a case of Horner’s syndrome each (63–66, 68). In the case series of 260 patients, there were 3 (1.15%) seromas, a wound infection (0.38%), and a tracheal fistula caused by ultrasonic scalpel cauterization (0.38%) (68).

#### 5.2.3 Advantages and limitations

A multidirectional approach with a symmetric view is an advantage of this technique, similar to a transcervical approach. Because this anatomy is more familiar to surgeons, some claim this technique is easier to adapt to (65). It has been shown to have comparable safety and oncologic outcomes compared to transcervical thyroidectomy and LND (65, 84). Multiple authors note the benefit of a robotic magnified view, making identification and preservation of parathyroids, nerves, and lymphatics easier (63, 69). Choi et al. hypothesized that the shorter LOS in the BABA could be attributed to less postoperative pain and the lower incidence of transient hypocalcemia in their study (69). When comparing the cost of BABA and a conventional transcervical procedure, the robotic procedure was almost 3-4 times more expensive (63, 69). This, and the longer operative times, are disadvantages to robotic remote access techniques, but the cosmetic benefit may be a reasonable tradeoff, especially for younger patients whose quality of life is significantly impacted by a large neck scar. Choi et al. note that due to the inferior-to-superior view, level IV dissection is challenging via this approach and requires adequate binding of lower breasts to change the pivot point (69). The larger area of dissection may also be a deterrent to choosing this approach, given the sensory changes associated. Further, the use of CO_2_ insufflation and possible complications, such as CO_2_ embolism, is another consideration.

### 5.3 Retroauricular (“Facelift”) approach

**Figure 6:**
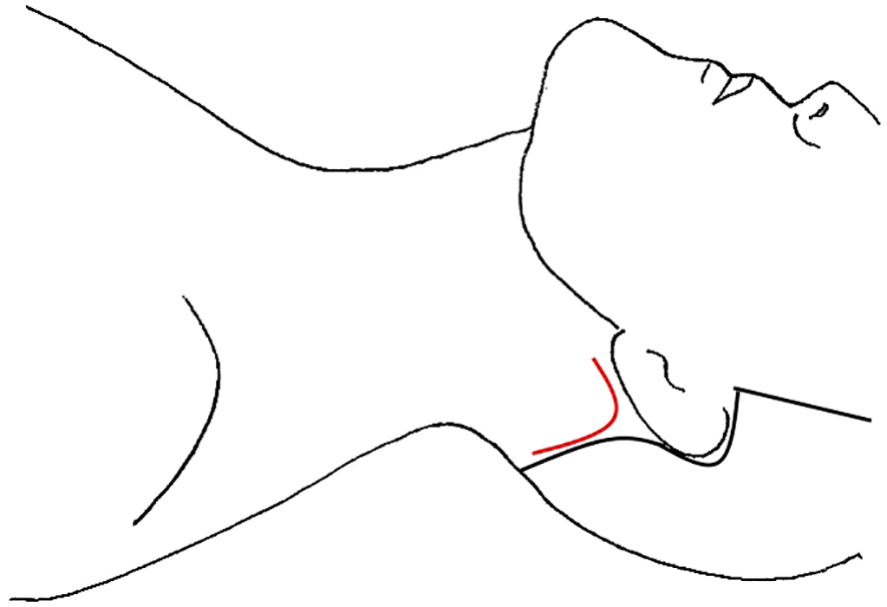
Retroauricular/Facelift Approach, incision location marked in red.

The unilateral retroauricular or modified “Facelift” approach (RAA) was first used in thyroid surgery by Terris et al. in 2011 and has since been expanded to include LND (23, 70, 71). While RAA is commonly used for neck dissection in other head and neck cancers, there were only two studies detailing its use for LND in thyroid cancer (**Table 8**) (85, 86). In Byeon et al’s case series, 4 female patients underwent total thyroidectomy and LND of II-V via unilateral RAA (70). The 15-patient case series by Lira et al. consisted of 12 patients who underwent RAA total thyroidectomy and LND II-V, and 3 patients who underwent LND only (71). In this technique, the incision is made in the postauricular crease, from the lower end of the retroauricular sulcus up to the midpoint and then curving to the occiput just inside the hairline (**Figure 6**). The flap is created below the platysma over the SCM to the midline, taking care to stay superior the greater auricular nerve and preserve it. The flap is extended to the sternal notch and clavicle inferiorly and submandibular gland superiorly. To expose the thyroid, the anterior inferior border of the SCM is dissected, as are the infrahyoid muscles off the thyroid. The strap muscles and flap are elevated with handheld retractors that are replaced with a self-retaining retractor (23, 70). Level II and III dissection is performed under direct vision, after which the robot is docked, and the camera and two working arms are inserted through the incision. Dissection of level IV and V is performed, with total thyroidectomy and CND to follow. A drain is typically left in the surgical field, exiting the incision, which is removed prior to discharge.

#### 5.3.1 Indications and contraindications

Indications for RAA include patients with DTC and LNM in the neck, no prior history of thyroid cancer, and no invasion of nearby structures. The contraindications were recurrence, extensive extrathyroidal spread or invasion of local structures, extranodal spread of LNM, and prior neck surgery or radiation (71).

#### 5.3.2 Outcomes and complications

Mean hospital LOS was 3.4 days in Brazil and 11 days in South Korea, which may speak to the health system differences more than morbidity of the surgery (70, 71). Mean operative times were 306.1 to 340 minutes, with the LND alone taking 153.1-251 min. Part of this increased time could be attributed to redocking the robotic arms for the LND, ipsilateral thyroidectomy, and contralateral thyroidectomy, respectively (70). Mean LN yield in the two series were 33.1 and 27.2, although the latter also included level VI (70, 71). All Byeon et al’s patients underwent RAI ablation and had low sTg at follow up (70). There was no evidence of recurrence in either study at mean follow up of 11.3-17.4 months.

Of the 19 patients total, there were 4 (21.05%) cases of transient hypocalcemia and 3 (15.79%) cases of transient RLN palsy, which all resolved by 3 months postoperative. There was 1 chyle leak in each series, one of which was managed conservatively and the other requiring reoperation. There was a total of 1 seroma, managed conservatively, and 1 wound infection. There were no cases of permanent hypocalcemia or RLN palsy. Lira et al. found their complication rates were comparable to MD Anderson Cancer Center’s published benchmarks for quality assessment in low acuity head and neck procedures (71).

#### 5.3.3 Advantages and limitations

When compared to other remote access techniques below the clavicles, this approach provides more direct access to the upper neck and requires a smaller area of dissection. As with other remote access techniques, patients avoid an anterior neck scar, with the cosmetic benefit being its biggest advantage. This approach can be done in higher BMI patients, and there no risk of brachial plexus or chest paresthesias, all of which are limitations to axillary approaches (21, 23). Because it is gasless, it avoids risks associated with insufflation. A limitation of this procedure is that most patients will experience hyperesthesia in the distribution of the greater auricular nerve which, while transient, can affect quality of life (23, 87). Another limitation is that performing a contralateral MRND is not possible from a unilateral approach and requires bilateral incisions, which may also be the case for contralateral lobectomy in some institutions, depending on surgeon comfort. Lastly, as with all robotic procedures, it is associated with increased cost.

### 5.4 Transaxillary and retroauricular approach

A bilateral postauricular and transaxillary approach was initially developed from BABA to eliminate incisions on the breast (88). A unilateral transaxillary and retroauricular approach (TARA) was later published to address the issue of accessing the upper neck via TAA alone, particularly level II and Va (73). There were two studies out of South Korea detailing LND via TARA, a case report and a comparative study of 22 TARA cases with 25 conventional transcervical cases (**Table 9**) (72, 73). In TARA, the retroauricular incision is made as described above in the retroauricular sulcus to the hairline in the occiput, and the subplatysmal flap is raised over the SCM. The spinal accessory nerve is identified and skeletonized along its course (73). The SCM is dissected and retracted laterally, allowing for direct vision dissection of levels Iib, Va, Iia, and the upper portion of III, in that order. Once complete, the 7-8cm incision in the anterior axillary line is made and the skin flap created above the pectoralis major as described in the TAA section. A self-retaining retractor is placed to elevate the flap and 3 robotic ports are inserted for the camera and two working arms. Dissection of level III, IV, and Vb proceed robotically via the transaxillary incision. It should be noted that the case report only performed dissection of levels III and IV via the transaxillary approach, while the 22 patients in the case series underwent MRND (excluding level I) as described here. Following completion of MRND, total thyroidectomy and CND are also completed through the transaxillary incision. This is aided by a small ipsilateral circumareolar incision for a 4^th^ robotic arm (73). A drain was left through the transaxillary incision and removed prior to discharge.

#### 5.4.1 Indications and contraindications

Indications for TARA included PTC with clinical LNM, no prior treatment for thyroid cancer, no extranodal spread of the LNM on pre-operative imaging, and cosmetic demand. Contraindications were recurrence, suspected extracapsular spread of LNM, or distant metastases (73).

#### 5.4.2 Outcomes and complications

When comparing 22 TARA and 25 transcervical cases of total thyroidectomy, CND, and MRND (levels II-V), Kim et al. reported no conversions to open (73). Mean LOS was 9.2 days and not significantly different between groups. Mean operative times were longer in the TARA group compared to the transcervical open group, 209.4 ± 38.2 vs 143.1 ± 30.5 minutes respectively, p=0.000. The only significant difference between groups was found on scar satisfaction scores, which were on a 5-point scale. The mean scar satisfaction score for the TARA group was 3.9 ± 1.0 vs. 2.8 ± 1.0 for the open group, p=0.000. The mean LN yield was 33.14 and similar to the open group, even when subdividing by each level. Both groups had similar sTg levels, and during the follow up period of 15.9 months, there were no recurrences (73).

There were no significant differences in complication rates. In the TARA group there were 6 (27.27%) cases of transient hypocalcemia, 2 (9.09%) transient RLN palsy, 1 (4.54%) hematoma, 2 (9.09%) seroma, and 1 (4.54%) chyle leak (73). All complications were managed conservatively, and the cases of transient hypocalcemia was resolved within 2 months. There were 6 patients who experienced postoperative earlobe numbness.

#### 5.4.3 Advantages and limitations

This approach has the advantage of avoiding an anterior neck scar and superior cosmetic satisfaction scores compared to transcervical conventional LND. The benefits of robotic surgery in terms of visualization and dexterity remain. Access to the upper neck levels is an advantage over other remote access techniques such as TAA or transoral. Many remote access LNDs do not include Va and IIb, however this approach includes those levels under direct vision. When comparing TARA to RAA or even TAA alone, the large dissection flap eliminates the advantage of a less invasive procedure. Contralateral MRND is not possible by this approach without performing bilateral TARA, which is a limitation. Further, this procedure is 7 times more expensive than a transcervical procedure in South Korea, which may be financially prohibitive for many patients (73).

### 5.5 Transoral vestibular approach

Robotic transoral thyroidectomy has also gained traction, although there are not many studies detailing LND via this approach. Tae et al. published a case report and case series of 14 patients (**Table 4**) (47, 49). In both studies, there were the standard three incisions in the oral vestibule, one 1.5-2cm in the midline near the frenulum and additional ports on either side laterally, close to the oral commissure (**Figure 3**), with standard access and initial creation of the subplastysmal space (47). Once the subplatysmal flap in the submental area is defined, the robot is docked, and the rest of the flap created robotically. To aid in retraction, a 4^th^ robotic arm is introduced through an additional 1 cm incision in the axilla on the proposed LND side. After total thyroidectomy and CND, SLND of level III-IV is performed in the standard fashion with preservation of the phrenic nerve, cervical roots, and transverse cervical artery. In the case series, if there were suspicious nodes in Vb, these were also dissected via the three vestibular incisions; however, 4 patients had suspected LNM in level II and required the addition of a gasless retroauricular approach (49). This incision is made in the postauricular sulcus and extends to the hairline in the occiput (**Figure 6**). The flap is created over the SCM under direct vision using electrocautery, until it connects with the prior surgical field, and the robot is docked with the camera and two working ports placed through the retroauricular incision (49). The working space is maintained via an external retractor through the retroauricular incision and level II is dissected robotically. A drain was left via the axillary incision in all cases and removed prior to discharge.

#### 5.5.1 Indications and contraindications

Indications for this approach included PTC with LNM in level II-V. Contraindications included a history of neck surgery or radiation, large fused LNM, extensive invasion of surrounding structures, recurrent disease, distant metastases, or extensive multi-level LNM (47, 49).

#### 5.5.2 Outcomes and complications

Mean operative time for the case series was 299 minutes for the transoral group and 431 minutes for the combined transoral and retroauricular approach. Thirteen patients underwent total thyroidectomy, CND, and SLND, while one patient had a lobectomy, CND, and SLND (49). One case was not completed via the transoral route but was converted to the retroauricular approach for uncontrolled IJV bleeding. The authors opted to convert to the gasless retroauricular approach rather than open transcervical due to strong patient preference to avoid a neck scar (49). The levels dissected varied (**Table 4**) but the mean LN yield was 30.7, similar to the case report yield of 29. Of the 10 patients that only underwent LND via the transoral approach, the mean LN yield was 23, while it was 38.3 for the combined approach (n=4) (49). Mean follow up time was 14.5 months and there were no recurrences on imaging studies.

There were no complications in the case report but the follow up time is also not specified. In the case series, 3 patients (21.43%) had transient hypocalcemia. There was 1 (7.14%) case each of chyle leak and transient RLN palsy. Four (28.57%) seromas occurred that resolved with repeated aspiration. There were no cases of permanent nerve injuries, hypocalcemia, wound infection, or hematoma (49).

#### 5.5.3 Advantages and limitations

Compared to the endoscopic transoral studies, the LN yield appears to be higher with the robotic approach, 23.1 ± 10.6 vs 10.9 ± 2.8 in Tan et al’s case series (n=20) (24). The robot is more ergonomic for the surgeon, with a 3-dimensional view of the surgical field and 7 degrees of freedom in wrist movement. The authors claim this makes flap creation, as well as superior pole dissection easier (49). In these studies, they also have the benefit of a third operative arm to aid in retraction, although this does require a small incision in the axilla. The biggest disadvantage to this approach is the increased time under anesthesia and costs associated with increased operative time and the use of the robot. Also, level II dissection is challenging without the additional postauricular incision.

## 6 Conclusion

This review summarizes the current literature regarding remote access lateral neck dissection for thyroid cancer. As demonstrated here, the limited data on these procedures is scattered across a variety of different approaches and techniques. For each approach, there are specific criteria for patient selection, but choice of technique ultimately depends on the surgeon’s training and facility with it. Importantly, remote access endoscopic and robotic neck dissections are being performed in specific high-volume centers by surgeons experienced in these procedures. Given the learning curve to perform these procedures and the need for a strong understanding of anatomical relationships, patients interested in remote access treatment for thyroid cancer should be referred to these high-volume centers to ensure the best surgical and oncological outcomes. The results outlined in this review should be interpreted in that context, and given the technical complexity of these procedures and limited number of facilities they are currently practiced in, they are unlikely to gain widespread adoption. Further, while there is debate in the literature on the effect of BMI on the technical challenges of creating skin flaps and adequate exposure, many of the remote access procedures are currently contraindicated in higher BMI patients, which limits their use in a large portion of the worldwide population. While short-term surgical outcomes and complication rates appear similar to conventional open lateral neck dissections based on the available literature, there is too little long-term data to draw conclusions about the oncologic outcomes in many of the approaches detailed above. As larger patient numbers and longer-term data become available, ongoing evaluation of the surgical and oncologic outcomes of these techniques is imperative.

## Author contributions

AW: Conceptualization, Writing – original draft, Writing – review & editing. MC: Writing – review & editing. CG: Conceptualization, Writing – review & editing.
