# Supplementary figures and images for: A scoping review of endoscopic and robotic techniques for lateral neck dissection in thyroid cancer
Source: Front Oncol. 2024 Feb 8;14:1297972. doi: 10.3389/fonc.2024.1297972 (PMC10883677; doi:10.3389/fonc.2024.1297972)

Supplemental Figure 1. Flow Chart of Review

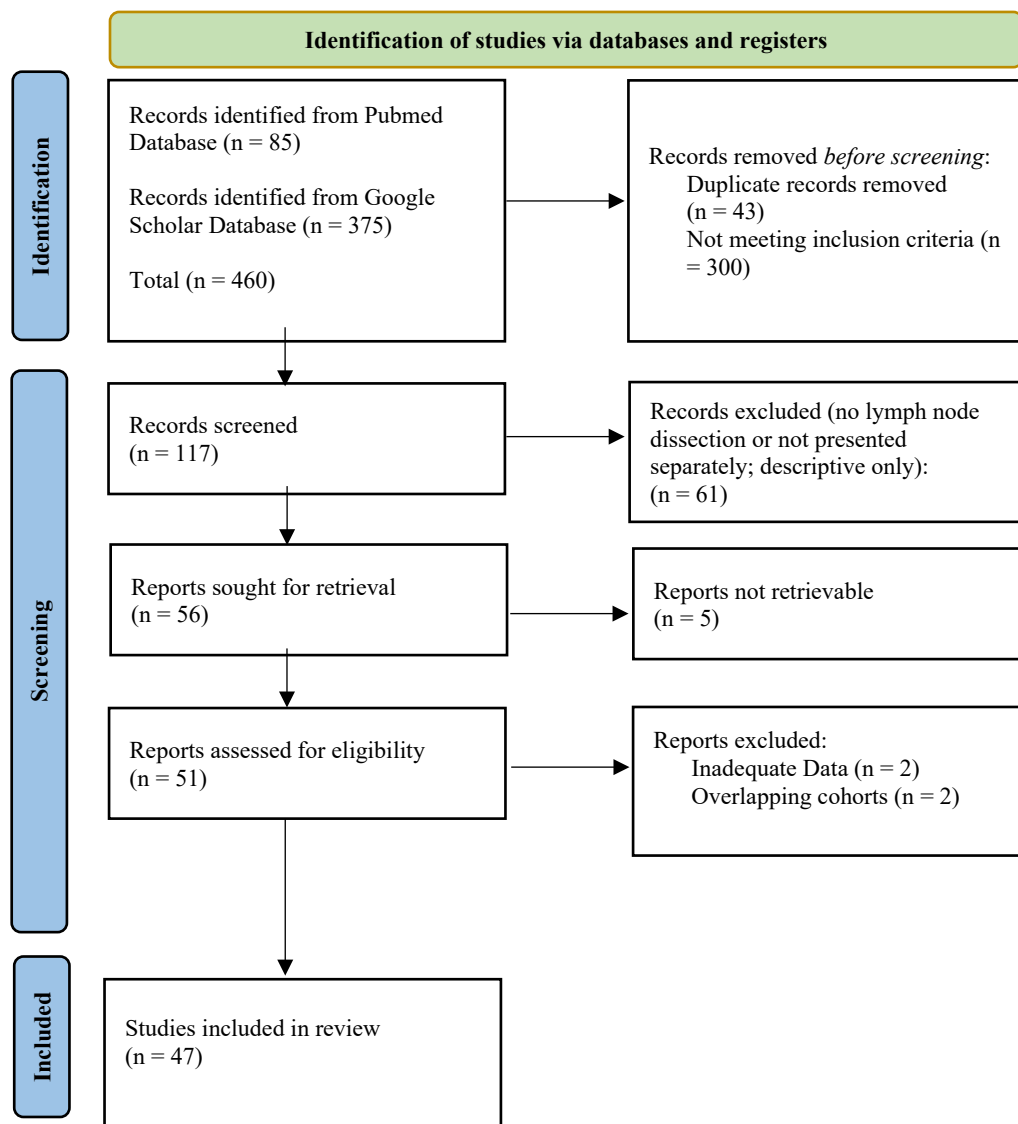

Supplement: Supplementary file 1 [file Image_1.pdf]
