# Supplementary material for: A scoping review of endoscopic and robotic techniques for lateral neck dissection in thyroid cancer
Source: Front Oncol. 2024 Feb 8;14:1297972. doi: 10.3389/fonc.2024.1297972 (PMC10883677; doi:10.3389/fonc.2024.1297972)
Supplement: Supplementary file 2 [file Table_1.docx]

| Supplemental Table 1: Methodological Items for Non-Randomized Studies | Methodological Items for  Non-Randomized Studies | | | | | | | | | Additional Criteria for Comparative Studies | | | | |  | |  | |
| --- | --- | --- | --- | --- | --- | --- | --- | --- | --- | --- | --- | --- | --- | --- | --- | --- | --- | --- |
| Author (Year of publication; citation number) | A clearly stated aim | Inclusion of consecutive patients | Prospective data collection | Endpoints appropriate to aim of study | Unbiased assessment of endpoints | Follow-up period appropriate | Loss to follow up <5% | Prospective calculation of study size | Adequate control group | | Contemporary groups | Baseline equivalence of groups | Adequate statistical analysis | Total | | Total Possible Points | |  |
| Ikeda et al (2002; 89) | 1 | 0 | 2 | 0 | 0 | 0 | 0 | 0 | N/A | | N/A | N/A | N/A | 3 | | 16 | |  |
| Lombardi et al (2007; 32) | 2 | 2 | 0 | 2 | 0 | 0 | 0 | 0 | N/A | | N/A | N/A | N/A | 6 | | 16 | |  |
| Miccoli et al (2008; 29) | 2 | 2 | 2 | 0 | 0 | 1 | 2 | 0 | N/A | | N/A | N/A | N/A | 9 | | 16 | |  |
| Wu et al (2013, 30) | 2 | 2 | 2 | 2 | 0 | 2 | 2 | 0 | N/A | | N/A | N/A | N/A | 12 | | 16 | |  |
| Zhang et al (2014; 31) | 1 | 1 | 0 | 0 | 0 | 0 | 0 | 0 | N/A | | N/A | N/A | N/A | 2 | | 16 | |  |
| Li et al (2016; 34) | 2 | 2 | 0 | 2 | 0 | 2 | 2 | 1 | 2 | | 2 | 0 | 2 | 17 | | 24 | |  |
| Zhang et al (2017; 33) | 2 | 2 | 0 | 2 | 0 | 2 | 2 | 0 | 2 | | 2 | 0 | 1 | 15 | | 24 | |  |
| Zhang et al (2017; 36) | 2 | 2 | 2 | 2 | 0 | 2 | 2 | 0 | 2 | | 2 | 0 | 1 | 17 | | 24 | |  |
| Xu et al(2020; 35) | 1 | 2 | 0 | 0 | 0 | 2 | 2 | 0 | 2 | | 2 | 0 | 1 | 12 | | 24 | |  |
| Ma et al (2022; 37) | 2 | 2 | 0 | 2 | 0 | 1 | 2 | 0 | 2 | | 2 | 0 | 2 | 15 | | 24 | |  |
| Kitagawa et al (2003; 40) | 2 | 2 | 0 | 0 | 0 | 0 | 0 | 0 | N/A | | N/A | N/A | N/A | 4 | | 16 | |  |
| Lin et al (2021; 39) | 2 | 2 | 2 | 2 | 0 | 2 | 2 | 0 | 2 | | 2 | 0 | 1 | 17 | | 24 | |  |
| Li et al (2011; 44) | 1 | 1 | 2 | 0 | 0 | 1 | 2 | 0 | N/A | | N/A | N/A | N/A | 7 | | 16 | |  |
| Yan et al (2015; 47) | 2 | 2 | 1 | 2 | 0 | 0 | 0 | 0 | N/A | | N/A | N/A | N/A | 7 | | 16 | |  |
| Guo et al (2019; 43) | 1 | 2 | 2 | 1 | 0 | 0 | 0 | 0 | 2 | | 2 | 1 | 0 | 11 | | 24 | |  |
| Wang et al (2019; 42) | 2 | 2 | 2 | 2 | 0 | 2 | 2 | 0 | N/A | | N/A | N/A | N/A | 12 | | 16 | |  |
| Yan et al (2021; 41) | 2 | 2 | 2 | 2 | 0 | 2 | 2 | 0 | 2 | | 2 | 2 | 0 | 18 | | 24 | |  |
| Chen et al (2022; 45) | 1 | 2 | 0 | 1 | 0 | 2 | 2 | 0 | N/A | | N/A | N/A | N/A | 8 | | 16 | |  |
| Tan et al (2020; 24) | 2 | 2 | 2 | 2 | 0 | 2 | 2 | 0 | N/A | | N/A | N/A | N/A | 12 | | 16 | |  |
| Tae et al (2020; 54) | 2 | 2 | 2 | 0 | 0 | 0 | 2 | 0 | N/A | | N/A | N/A | N/A | 8 | | 16 | |  |
| Ngo et al (2021; 52) | 2 | 2 | 2 | 0 | 0 | 0 | 0 | 0 | N/A | | N/A | N/A | N/A | 6 | | 16 | |  |
| Tae et al (2022; 88) | 2 | 2 | 2 | 2 | 0 | 2 | 2 | 0 | N/A | | N/A | N/A | N/A | 12 | | 16 | |  |
| Kuang et al (2022; 46) | 2 | 2 | 0 | 2 | 0 | 2 | 2 | 0 | N/A | | N/A | N/A | N/A | 10 | | 16 | |  |
| Chen et al (2022; 18) | 2 | 2 | 2 | 2 | 0 | 2 | 2 | 0 | N/A | | N/A | N/A | N/A | 12 | | 16 | |  |
| Wang et al (2023; 56) | 2 | 2 | 0 | 2 | 0 | 2 | 2 | 0 | N/A | | N/A | N/A | N/A | 10 | | 16 | |  |
| Wang et al (2023; 55) | 2 | 2 | 2 | 1 | 0 | 1 | 2 | 0 | 0 | | 2 | 2 | 0 | 14 | | 24 | |  |
|  |  | | | | | | | |  | | | | |  | |  | |  |
|  | Methodological Items for  Non-Randomized Studies | | | | | | | | Additional Criteria for Comparative Studies | | | | |  | |  | |  |
| Author (Year of publication; citation number) | A clearly stated aim | Inclusion of consecutive patients | Prospective data collection | Endpoints appropriate to aim of study | Unbiased assessment of endpoints | Follow-up period appropriate | Loss to follow up <5% | Prospective calculation of study size | Adequate control group | | Contemporary groups | Baseline equivalence of groups | Adequate statistical analysis | Total | | Total Possible Points | |  |
| Kang et al (2009; 65) | 1 | 2 | 2 | 2 | 0 | 2 | 2 | 0 | N/A | | N/A | N/A | N/A | 11 | | 6 | |  |
| Kang et al (2010; 62) | 1 | 2 | 0 | 1 | 0 | 2 | 2 | 0 | N/A | | N/A | N/A | N/A | 8 | | 16 | |  |
| Kang et al (2012; 63) | 2 | 2 | 0 | 1 | 0 | 2 | 1 | 0 | 2 | | 2 | 0 | 0 | 12 | | 24 | |  |
| Yoon et al (2013; 64) | 2 | 2 | 0 | 0 | 0 | 2 | 1 | 2 | 2 | | 2 | 2 | 0 | 15 | | 24 | |  |
| Lee et al (2013; 59) | 2 | 2 | 2 | 2 | 0 | 2 | 2 | 0 | 2 | | 2 | 1 | 0 | 17 | | 24 | |  |
| Song et al (2015; 57) | 2 | 2 | 2 | 2 | 0 | 2 | 2 | 0 | N/A | | N/A | N/A | N/A | 12 | | 16 | |  |
| Song et al (2016; 58) | 2 | 2 | 0 | 2 | 0 | 2 | 2 | 2 | 2 | | 2 | 0 | 0 | 16 | | 24 | |  |
| Garstka et al (2018; 66) | 2 | 2 | 2 | 2 | 0 | 2 | 2 | 0 | 2 | | 2 | 0 | 0 | 16 | | 24 | |  |
| Kim et al (2018; 61) | 2 | 2 | 0 | 2 | 0 | 2 | 2 | 0 | N/A | | N/A | N/A | N/A | 10 | | 16 | |  |
| Kim et al (2022; 60) | 2 | 2 | 0 | 2 | 0 | 0 | 0 | 0 | N/A | | N/A | N/A | N/A | 6 | | 16 | |  |
| Seup Kim et al (2015; 73) | 2 | 2 | 0 | 2 | 0 | 2 | 2 | 0 | 2 | | 2 | 1 | 0 | 15 | | 24 | |  |
| Choi et al (2017; 72) | 0 | 0 | 0 | 1 | 0 | 2 | 2 | 0 | N/A | | N/A | N/A | N/A | 5 | | 16 | |  |
| Yu et al (2018; 75) | 2 | 2 | 0 | 0 | 0 | 2 | 2 | 0 | N/A | | N/A | N/A | N/A | 8 | | 16 | |  |
| Paek et al (2020; 77) | 2 | 2 | 0 | 2 | 0 | 0 | 0 | 0 | 2 | | 2 | 2 | 0 | 12 | | 24 | |  |
| Song et al (2020; 74) | 2 | 0 | 0 | 2 | 0 | 2 | 2 | 0 | N/A | | N/A | N/A | N/A | 8 | | 16 | |  |
| He et al (2020; 76) | 2 | 2 | 0 | 2 | 0 | 2 | 2 | 0 | N/A | | N/A | N/A | N/A | 10 | | 16 | |  |
| Choi et al (2021; 51) | 2 | 2 | 0 | 2 | 0 | 0 | 0 | 0 | 2 | | 2 | 0 | 0 | 10 | | 24 | |  |
| Byeon et al (2014; 81) | 2 | 2 | 2 | 0 | 0 | 2 | 2 | 0 | N/A | | N/A | N/A | N/A | 10 | | 16 | |  |
| Lira et al (2018; 80) | 2 | 2 | 0 | 2 | 0 | 2 | 2 | 0 | N/A | | N/A | N/A | N/A | 10 | | 16 | |  |
| Byeon et al (2012; 87) | 2 | 1 | 0 | 2 | 0 | 0 | 0 | 0 | N/A | | N/A | N/A | N/A | 5 | | 16 | |  |
| Kim et al (2014; 86) | 2 | 2 | 2 | 2 | 0 | 2 | 2 | 0 | 2 | | 2 | 0 | 0 | 16 | | 24 | |  |
